# Window of opportunity to achieve major outcomes in early rheumatoid arthritis patients: how persistence with therapy matters

**DOI:** 10.1186/s13075-015-0697-z

**Published:** 2015-07-11

**Authors:** Irazú Contreras-Yáñez, Virginia Pascual-Ramos

**Affiliations:** Department of Immunology and Rheumatology, Instituto Nacional de Ciencias Médicas y Nutrición Salvador Zubirán, Vasco de Quiroga 15, Colonia Belisario Domínguez Sección XVI, 14080 Tlalpan, DF Mexico

## Abstract

**Introduction:**

Benefits of disease-modifying anti-rheumatic drugs (DMARD) in early rheumatoid arthritis patients (ERAP) will be achieved if patients follow prescribed treatment. Objective was to investigate whether timing of first non-persistence period and/or duration of persistence during the first 4 years of follow-up predicted disease outcomes at the 5^th^ year in a cohort of ERAP, initiated in 2004.

**Patients and Methods:**

Up to February 2015, charts of 107 ERAP with at least 5 years of follow-up and prospective 6-month assessments of disease activity, disability and persistence were reviewed. Non-persistence was defined as omission of DMARD and/or corticosteroids for at least 7 consecutive days; regarding methotrexate, one weekly missing dose was considered non-persistence. Persistence was recorded through an interview (up to 2008) and thereafter through a questionnaire; persistence duration was recorded in months of continuous medicationtaking. At the 5^th^ year, disease activity was defined according to Disease Activity Score (DAS)28, and disability according to Health Assessment Questionnaire (HAQ). Descriptive statistics and linear and Cox regression analyses were used.

**Results:**

At study entry, patients were more frequently middle-aged (39.1 ± 13.3 years) and female (88.8 %), as well as more likely to have high disease activity and disability. Over the first 4 years of follow-up, 54.2 % of the patients had indications for oral corticosteroids and all traditional DMARDs. Almost 70 % had at least one period of non-persistence, and their follow-up (median, 25th–75th interquartile range) to first non-persistence period was 13 months (1–31). Persistence duration during the first 4 years predicted subsequent DAS28 (in addition to gender and baseline DAS28) and HAQ (in addition to age). During the 5^th^ year, 68 patients (56 women) achieved sustained remission (DAS28 < 2.6). In female population (*n* = 95), baseline DAS28 (odds ratio [OR], 0.65; 95 % confidence interval [CI], 0.50–0.83; *p* = 0.001) and persistence duration (OR, 1.04; 95 % CI, 1–1.08; *p* = 0.05) were predictors. Also, 84 patients achieved sustained function (HAQ <0.21), and baseline DAS28 and age were the only predictors. Timing of first non-persistence period did not impact outcomes.

**Conclusions:**

Persistence duration with DMARDs within the first 4 years of RA predicted subsequent favorable outcomes in ERAP; additional predictors were younger age, male gender and lower disease activity at diagnosis.

## Introduction

The window of opportunity concept states that there are superior clinical responses and the potential for remission when patients with rheumatoid arthritis (RA) are managed early and aggressively with disease-modifying anti-rheumatic drugs (DMARDs) [1–3]. Early RA clinics are the perfect setting to address such concept, as patients with recent disease onset are referred to experts in rheumatologic care without delay. Nonetheless, it seems intuitive to assume that the full benefit of the pharmacologic intervention will be achieved if patients follow prescribed regimens reasonably closely.

*Medication adherence* refers to the patient’s act of conforming to the recommendations made by the health care provider with respect to timing, dosage and frequency of medication-taking [4, 5]. Medication adherence can be divided into three major components: (1) *persistence*, defined as the length of time a patient fills prescriptions without permissible gaps; (2) *initiation adherence*, defined as the patient starting with the intended treatment; and (3) *execution adherence* that results from the comparison between the prescribed drug regimen and the patient’s actual drug-taking behavior [4].

Poor adherence with therapy affects 20 to 70 % of patients with RA, sometimes during their follow-up [6–14]. Local experience within a cohort of Mexican Mestizo patients with early RA (ERAP) confirmed these data and additionally showed that poor adherence with traditional DMARDs was associated with increased disease flares, decreased rates of remission and worse patient-reported outcomes [15–17], although the mechanisms were not examined. In particular, there may be aspects within the adherence and persistence constructs, such as duration (of persistence) or timing (of first non-persistence period), that may have a different (and/or additive) impact on outcomes. We sought to explore this hypothesis in a well-defined population of ERAPs in whom persistence was prospectively evaluated and the concept of window of opportunity was extended to the construct of persistence.

The following were the specific objectives of the study:

1. To investigate if the timing of first non-persistence period and/or duration of persistence during the first 4 years of follow-up were predictors of the level of disease activity at the fifth year of follow-up and of achieving sustained remission (SR).
2. If the first objective was achieved, we planned to extend the analysis to a patient-reported outcome and investigate a similar impact on patient function as evaluated per the Health Assessment Questionnaire (HAQ) [18].
3. To define additional predictors of disease outcomes.

## Methods

### Setting and study population

The Instituto Nacional de Ciencias Médicas y Nutrición Salvador Zubirán is a national referral center for rheumatic diseases that belongs to the National Institutes of Health in Mexico City. In 2004, an early arthritis clinic was established. Patients entering the clinic had disease durations of less than 1 year when first evaluated and no specific rheumatic diagnosis other than RA. Patients were evaluated every 2 months during the first 2 years of follow-up and thereafter every 2, 4 or 6 months (fixed for all the patients from the baseline evaluation), depending on patient and disease characteristics. Treatment was prescribed by the rheumatologist in charge of the clinic and was given using a treat-to-target (T2T) approach. Briefly, at every medical encounter, the Disease Activity Score in 28 joints (DAS28) was calculated and the level of disease activity defined. If remission was achieved, no major treatment modifications were made; if not, treatment was intensified with the aim of achieving remission, which was defined as DAS28 less than 2.6. Traditional DMARDs were used in 99 % of our population, with or without corticosteroids (around 50 % of the patients). From the beginning of the clinic, patients’ medication behavior was prospectively assessed, initially through a structured interview and starting in November 2008 by using an instrument locally designed, the Concordance Questionnaire (CQ) formerly called the Compliance Questionnaire, which evaluates both constructs: adherence to and persistence with therapy [16].

Up to February 2015, 165 ERAPs had been evaluated, of whom 107 had at least 5 years of follow-up (19 patients were lost to follow-up, and 2 additional patients died). The 5 years of follow-up was deemed to be sufficient to accomplish the objectives described.

### Rheumatic evaluations

At study entry, a complete medical history and demographic data, along with disease-specific autoantibodies, were recorded. Medical evaluations were standardized and included swollen and tender joint counts, acute reactant-phase determinations, patient- and physician- reported outcomes, comorbidity established by record review and treatment assessment (names, doses and schedules of all drugs taken since last visit), along with the evaluation of persistence.

### Persistence evaluation

From 2004 to 2008, persistence was assessed through an interview conducted at every visit by the same rheumatologist. Patients were directed to report the names, doses and schedules of DMARDs and corticosteroids they had taken since last visit (fixed at 6 months apart), initially spontaneously and if necessary directly. Then, patients were asked about any missing and/or incorrect medications, doses and/or schedules since their last visit. The number of days of missing medication was recorded. The rheumatologist compared the last prescription and the actual treatment, and, if inconsistencies were found, they were resolved. Data were collected in standardized formats.

Since 2008, persistence (and adherence) was evaluated through the CQ. The performance of the CQ has shown high sensitivity and satisfactory specificity to detect persistence [16]. CQ was fulfilled without help by 95 % of the patients.

### Definitions

*Sustained remission* was assessed at the fifth year of follow-up and defined if DAS28 was below 2.6 at the three consecutive evaluations within that year [19, 20].

The DAS28 at the fifth year of follow-up was calculated as the mean of individual DAS28 from visits performed during the fifth year.

*Patient function* was also evaluated at the fifth year of follow-up. A patient was considered to be without disability if the HAQ score was sustained at or below 0.20 at all consecutive evaluations within that year. In addition, HAQ score at the fifth year was derived from the mean of individual HAQs from visits during the fifth year.

According to the interview, *non-persistence* with medication was defined as omission of at least one DMARD and/or corticosteroid for at least 7 consecutive days. Regarding methotrexate, at least one missing weekly dose was considered non-persistence. Treatment modifications because of adverse events and/or indicated by a different physician for any reason (e.g., insufficient response, pregnancy, schedule surgery) were not considered non-persistence under the construct. According to the CQ, a patient was considered to be non-persistent if, in item 10 (“In the past 6 months, how often did you completely stop taking your DMARDs?”), boxes 2 (“Sometimes”), 3 (“Almost always”) and 4 (“Always”) were filled. Persistence was evaluated at 6-month periods (fixed for all the patients) and defined by an independent observer according to the information recorded in standardized formats.

### Ethics

The study was approved by the institution’s internal review board (*Comites de Ética e Investigación del Instituto Nacional de Ciencias Médicas y Nutrición Salvador Zubirán*)*.* Written informed consent was obtained from all the patients when entering the clinic. Also, specific written consent was obtained to have each patient’s charts reviewed and data presented in scientific forums or publications.

### Statistical analysis

Descriptive statistics, Student’s *t* test and *χ*^2^ test were used as appropriate. Sociodemographic data are presented as mean ± standard deviation (SD), and disease and treatment characteristics are described as median and 25th–75th interquartile range (IQR).

For each patient, persistence was evaluated at fixed 6-month intervals during the first 4 years of follow-up, with a total of eight consecutive persistence evaluations (persistence 1 to persistence 8); accordingly, for each patient, duration of persistence varied from 0 to 48 months.

Also, for each patient, the timing of (or follow-up to) first non-persistence period was obtained and scored as 1 month (if first non-persistence was detected at persistence 1 evaluation), 7 months (if first non-persistence was detected at persistence 2 evaluation), 13 months (if first non-persistence was detected at persistence 3 evaluation), 19 months (if first non-persistence was detected at persistence 4 evaluation) and successively (adding 6 months to each consecutive persistence evaluation) up to 43 months if first non-persistence was detected at the persistence 8 evaluation.

Linear regression analysis was used to investigate the impact of the timing of the first non-persistence period and the duration of persistence during the first 4 years of follow-up (independent variables) on DAS28 and HAQ at the fifth year of follow-up (dependent variables). Also, Cox regression analysis was used to investigate whether the timing of the first non-persistence period and the duration of persistence during the first 4 years of follow-up (independent variables) made a contribution to SR and sustained function (SF) at the fifth year of follow-up (dependent variables). Variables included in the different models tested were selected based on their statistical significance in the univariate analysis and also on their clinical relevance (e.g., age and comorbidity). In particular, age was forced into the models based on previous local report, where it has been found a predictor of poor adherence and subsequently worse outcomes [15]. Correlation between variables to be included was also examined, and the final number of variables was limited by the number of outcomes of interest. Significant variables were finally isolated using stepwise selection. Analysis was repeated for each outcome and in the subpopulation of female patients with RA.

All statistical tests were two-sided and evaluated at the 0.05 significance level. Statistical analysis was performed using the IBM SPSS software program (v.17.0; IBM, Armonk, NY, USA).

## Results

### Characteristics of the study population

Charts from 107 ERAPs were reviewed, and their data are summarized in Table 1. Patients were predominantly female (88.8 %), middle-aged (mean ± SD was 39.1 ± 13.3 years), with 11.1 ± 3.9 years of formal education. Nine patients (8.4 %) were current smokers when they entered the clinic. At the baseline evaluation, 88 patients (82.2 %) had rheumatoid factor (RF) and 92 (86 %) had anti-cyclic citrullinated peptide antibodies. All had recent-onset disease (median [IQR] disease duration of 5 months [3.4–7] and high disease activity [DAS28 of 6 [5.1–7.1]) and substantial disability (median [IQR] HAQ of 1.5 [0.9–2.1]). Eleven patients (10.3 %) had erosive disease, and 89 (77.4 %) had at least one comorbid condition.

**Table 1:** Population characteristics and comparison of patients with versus without sustained remission

	Whole population (*N* = 107)	Patients with SR (*n* = 68)	Patients without SR (*n* = 39)	*p* Value^a^
Sociodemographic variables				
Female sex, *n* (%)	95 (88.8)	56 (82.4)	39 (100)	0.004
Age at baseline, years, (mean ± SD)	39.1 ± 13.3	38.6 ± 13.7	39.1 ± 13.4	0.85
Years of formal education, (mean ± SD)	11.1 ± 3.9	13.4 ± 4.1	8.6 ± 3	0.04
Current smokers, *n* (%) of patients	9 (8.4)	7 (10.3)	2 (22.2)	0.48
Disease characteristics at baseline^b^				
Disease duration, months	5 (3.4–7)	4.2 (2.9–4.9)	4.9 (2.6–6.2)	0.22
Patients with RF, *n* (%)	88 (82.2)	54 (79.4)	34 (87.2)	0.43
Patients with ACCP, *n* (%)	92 (86)	57 (83.8)	35 (89.7)	0.57
DAS28	6 (5.1–7.1)	5.7 (4.6–6.7)	7 (6.1–7.8)	0.001
HAQ	1.5 (0.9–2.1)	1.3 (0.6–2)	1.9 (1.5–2.4)	0.000
Patients with erosions, *n* (%)	11 (10.3)	8 (11.8)	3 (7.7)	0.74
Number (%) of patients with ≥1 comorbidity	89 (77.4)	57 (83.8)	28 (71.8)	0.15
Number of comorbidities/patient	1 (1–2)	1 (1–2)	2 (1–2.8)	0.08
Cumulative treatment characteristics^b^				
Patients with corticosteroids, *n* (%)	58 (54.2)	33 (48.5)	25 (64.1)	0.16
Number of DMARDs/patient	2.2 (1.9–2.9)	2.2 (1.7–2.8)	2.6 (2–3)	0.03
Number (%) of patients with ≥1 non-persistence period	74 (69.2)	46 (67.6)	28 (71.8)	0.83
Follow-up at first non-persistence period,^c^ mo	13 (1–31)	19 (7–31)	7 (1–23.5)	0.02
Persistence duration, mo^d^	42 (30–48)	42 (30–48)	36(18–48)	0.07

*ACCP* Anti-cyclic citrullinated peptide antibodies, *DAS28* Disease Activity Score in 28 joints, *DMARD* Disease-modifying anti-rheumatic drug, *HAQ* Health Assessment Questionnaire, *RF* Rheumatoid factor, *SD* Standard deviation, *SR* Sustained remission

^a^ *p* < 0.05 is statistically significant

^b^Data are presented as median (25th–75th interquartile range) unless otherwise indicated

^c^Restricted to 74 patients with ≥1 non-persistence period

^d^In the whole population

During the 4-year follow-up, 58 patients (54.2 %) had indications for oral corticosteroids at some point, and all patients had indications for DMARDs. The median (IQR) number of DMARDs per patient was 2.2 (1.9–2.9). Table 2 summarizes treatment at baseline and at last follow-up of the evaluation of the persistence period. Also, 74 patients (69.2 %) had at least one period of non-persistence, and their follow-up to first non-persistence period was 13 months (1–31). Finally, the whole population had a median (IQR) of 42 (30–48) months of persistence during follow-up evaluated.

**Table 2:** Treatment strategies at baseline and last follow-up

Treatment strategies, *n* (%) of patients with	At baseline evaluation	At last persistence evaluation
Oral corticosteroids	42 (36.5)	48 (41.7)
Methotrexate monotherapy	20 (17.4)	43 (37.4)
2 combined DMARDs (methotrexate required)	68 (59.1)	40 (34.8)
≥3 combined DMARDs (methotrexate required)	21 (18.3)	22 (19.1)
Other combinations of traditional DMARDs	6 (5.2)	10 (8.7)
Biologic DMARDs	0	5 (4.3)

*DMARDs* disease-modifying anti-rheumatic drugs

### Predictors of disease activity and sustained remission (sustained remission at the fifth year of follow-up)

During the fifth-year follow-up, 68 patients (63.6 %) achieved SR and 39 (26.4 %) did not. Table 1 summarizes these differences. Patients in the former group were less frequently female, had more years of formal education, had lower disease activity and disability at baseline, had indications for fewer DMARDs per patient during follow-up and tended to have a higher persistence duration and more comorbidities per patient. Also, among the restricted population with at least one non-persistence period (*n* = 74), patients from the former group had longer follow-up to first non-persistence period.

To determine predictors of DAS28 at the fifth year follow-up, linear regression models were applied. The following variables were entered in the model: sex, education, baseline DAS28 (highly correlated to HAQ, ρ = 0.68, *p* ≤ 0.001), DMARDs per patient, timing of non-persistence and persistence duration. In some models, age and comorbidities per patient were forced. As shown in Table 3, sex, baseline DAS28 and persistence duration predicted DAS28 at the fifth year, and the strongest impact was due to months of persistence. When the model was tested in the subpopulation of women, DAS28 and persistence duration were still the only predictors of disease activity at the fifth year (data not shown).

**Table 3:** Linear regression models to predict DAS28 and HAQ score at the fifth year of follow-up

Variables^a^	DAS28 at the fifth year^b^	HAQ at the fifth year^c^
Male sex	0.64, (0.005 to 1.28), 0.05	
Baseline DAS28	0.24, (0.09 to 0.38), 0.002	
Months of persistence duration during the first 4 years	−0.28, (−0.045 to −0.011), 0.002	−0.12, (−0.019 to −0.005), 0.001
Age		0.10, (0.004 to 0.016), 0.001

*DAS28* Disease Activity Score in 28 joints, *HAQ* Health Assessment Questionnaire

^a^Data are presented as β coefficients (95 % confidence interval), *p* value

^b^ *R* ^2^ = 0.338

^c^ *R* ^2^ = 0.281

We then performed Cox regression analysis to determine predictors of achieving SR at the fifth year, and the variables described above were considered in different models tested. DAS28 at baseline (odds ratio [OR], 0.65; 95 % confidence interval [CI], 0.50–0.83; *p* ≤ 0.001), male sex (OR, 0.42, 95 % CI, 0.18–0.97; *p* = 0.04) and persistence duration within the first 4 years of follow-up (OR, 1.03, 95 % CI, 1–1.07; *p* = 0.06) were the only predictors of SR at the fifth year. We repeated the analysis in the female subpopulation. Comparison of women with versus without SR showed similar results to those described in Table 1 (data not shown). In the Cox analysis, baseline DAS28 (OR, 0.65; 95 % CI, 0.50–0.83; *p* = 0.001) and persistence duration (OR, 1.04; 95 % CI, 1–1.08; *p* = 0.05) still predictors.

**Fig. 1:**
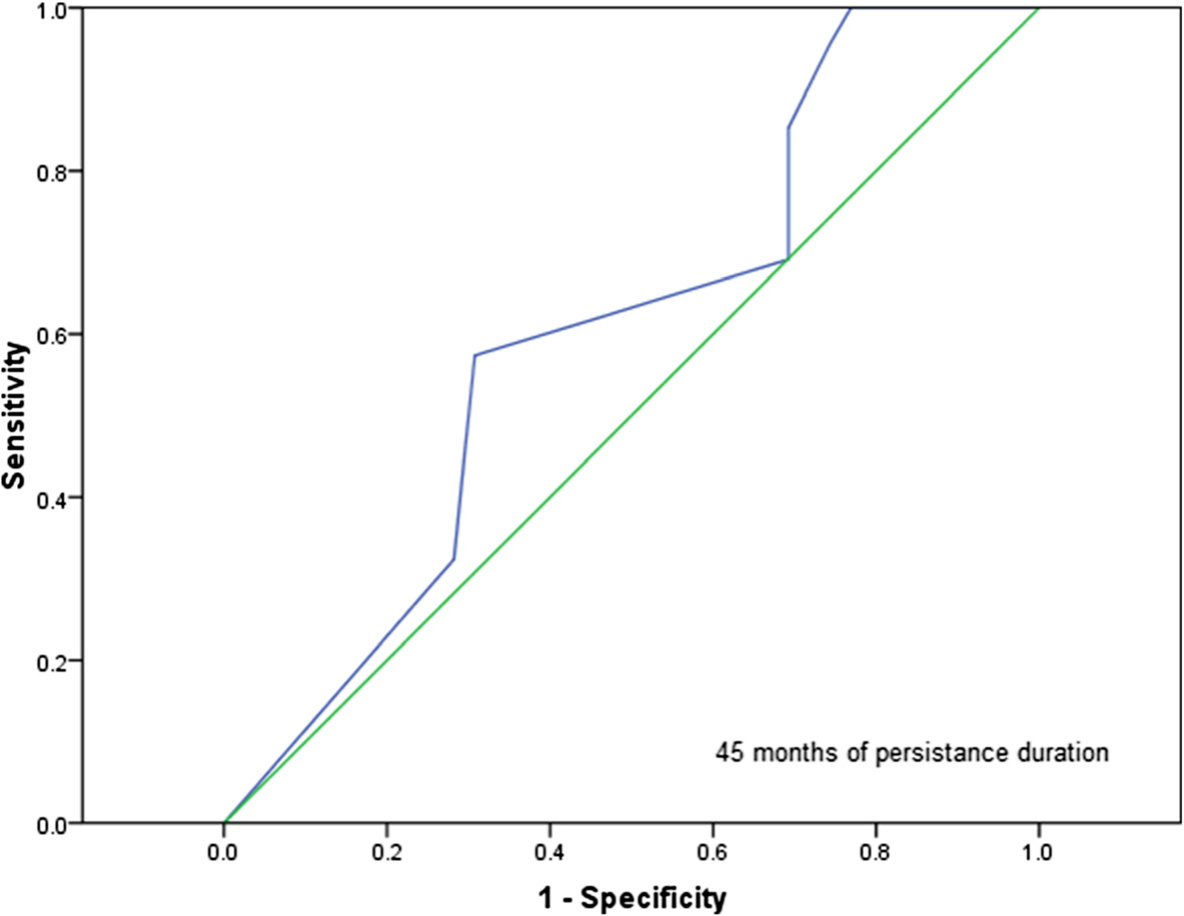
Receiver operating characteristic curve for cutoff for persistence duration to predict sustained remission. Curve plots the relationship between sensitivity and specificity for the persistence duration cutoff to predict SR at the fifth year of follow-up

In the subpopulation of women, according to receiver operating characteristic curve analysis, the best cutoff for persistence duration (during the first 4 years of follow-up) to predict SR at the fifth year was 41.5 months (sensitivity, 0.64; specificity, 0.64; area under the curve, 0.67; 95 % CI, 0.54–0.79) (Fig. 1).

### Predictors of Health Assessment Questionnaire score and sustained function at the fifth year of follow-up

During the fifth year of follow-up, 84 patients (78.5 %) achieved SF according to our definition and 23 (21.5 %) did not. Table 4 summarizes differences between these groups. Those who achieved SF were younger, tended to be more educated, had more disease duration at diagnosis (although both had recent-onset disease), had lower disease activity and disability at the baseline evaluation and tended to have higher persistence duration.

**Table 4:** Comparison of patients with versus without sustained function

	Patients with SF at fifth year, *n* = 84	Patients without SF at fifth year, *n* = 23	*p* Value^a^
Sociodemographic variables			
Female sex, number (%) of patients	73 (86.9)	22 (95.7)	0.46
Age at baseline, yr (mean ± SD)	36.1 ± 12.2	48.5 ± 13.6	0.000
Years of formal education (mean ± SD)	11.3 ± 3.9	9.7 ± 4.2	0.09
Current smokers, number (%) of patients	9 (10.7)	0 (0)	0.2
Disease characteristics at baseline^b^			
Disease duration, mo	5.3 (3.8–7.5)	3.8 (2.5–6.2)	0.05
Patients with RF, *n* (%)	69 (82.1)	19 (82.6)	1
Patients with ACCP, *n* (%)	72 (85.7)	20 (87)	1
DAS28	6 (4.9–6.9)	6.8 (6–7.7)	0.02
HAQ	1.4 (0.8–2)	2.1 (1.6–3)	0.000
Patients with erosions, *n* (%)	9 (10.7)	2 (8.7)	1
Number (%) of patients with ≥1 comorbidity	65 (77.4)	20 (87)	0.39
Number of comorbidities/patient	1 (1–2)	2 (1–2.8)	0.2
Cumulative treatment characteristics^b^			
Patients with corticosteroids, *n* (%)	42 (50)	16 (69.6)	0.11
Number of DMARDs/patient	2.3 (1.9-2.9)	2.4 (2–3)	0.44
Number (%) of patients with ≥1 non-persistence period	57 (76.9)	17 (73.9)	0.8
Follow-up at first non-persistence period,^c^ mo	13 (1–31)	13 (1–22)	0.25
Persistence duration, mo^d^	42 (30–48)	36 (12–48)	0.08

*ACCP* anti-cyclic citrullinated peptide antibodies, *DAS28* Disease Activity Score in 28 joints, *DMARDs* disease-modifying anti-rheumatic drugs, *HAQ* Health Assessment Questionnaire, *RF* rheumatoid factor, *SD* standard deviation, *SF* sustained function

^a^ *p* < 0.05 is statistically significant

^b^Data presented as median (25th–75th interquartile range) unless otherwise indicated

^c^Restricted to 74 patients with ≥1 non-persistence period

^d^In the whole population

To determine predictors of HAQ at fifth year, linear regression models were applied. The following variables were entered in the model: age, disease duration, baseline DAS28 (highly correlated to baseline HAQ; ρ = 0.68, *p* ≤ 0.001) and persistence duration. Additional variables forced into the model were treatment, timing of non-persistence and comorbidities per patient. As shown in Table 3, age and persistence duration predicted HAQ at the fifth year. Similar results were obtained when the model was applied to the female subpopulation (data not shown).

We then performed Cox regression analysis to determine predictors of achieving SF at the fifth year, and the variables described above were considered in different models tested. DAS28 at baseline (OR, 0.79; 95 % CI, 0.66–0.94; *p* = 0.01) and age (OR, 0.97; 95 % CI, 0.95–0.99; *p* = 0.002) were the only predictors of SR at the fifth year.

Finally, all of the above analyses were repeated in the population with at least one non-persistence period (*n* = 74), and similar results were obtained. Also, there was a moderate correlation between duration of persistence and follow-up to first non-persistence period (ρ = 0.46, *p* ≤ 0.001). When persistence duration was switched in models of follow-up to first non-persistence period, only variables unrelated to the persistence construct prevailed.

## Discussion

This study was developed in a well-characterized cohort of Mexican Mestizo patients with early RA disease and substantial comorbidity. Standardized and complete follow-up was performed by the same rheumatologist in a real clinical setting. Follow-up included periodic and prospective evaluations of comorbidity and of treatment persistence. Conventional DMARDs given according to a T2T strategy were the mainstay of treatment, and a substantial follow-up was included. For all these reasons, we consider the population described to be representative of “real-life patients”, so the results presented here have clinical and practical implications and can be generalized to populations with similar characteristics.

The duration of persistence with DMARDs during the first 4 years of follow-up was a predictor of disease activity at the consecutive year (in addition to baseline disease activity and sex) and of disability (in addition to age). Also, persistence duration during the first 4 years (along with lower baseline disease activity) predicted SR at the consecutive year in women. The timing of first non-persistence period (early vs. late) did not impact disease outcomes. Forty-two months of persistence within the first 4 years of follow-up that corresponded to 87.5 % of the complete potential persistence length was the best cutoff for persistence duration to predict SR in the subpopulation of women, who were highly represented in our cohort. Finally, lower disease activity and younger age were the only predictors of SF.

Our study confirms prior literature reviews which highlighted that adherence to and persistence with traditional and biologic DMARDs among patients with RA are suboptimal [20–23]. Persistence impacted outcomes in our population of early RA patients. Viller et al. also showed better outcomes in European patients with early RA (≤5-year disease duration) in whom compliance with drug dosages and dosing times was assessed yearly using a questionnaire [13]. We recently showed, in the same inception cohort, better outcomes in adherent and persistent patients in two different clinical scenarios: patients with high disease activity and disability and patients in remission or who had low disease activity [15, 16]. The present study adds additional information to the field of the burden of inadequate therapeutic behavior in patients with RA patients. In addition to our examination of sustained and major outcomes, we show that the duration of persistence impacted disease outcomes, whereas the timing of non-persistence did not. Also, in the female subpopulation, a substantial persistence duration with DMARDs was required to achieve SR (almost 88 % of the entire length of persistence). Several studies have shown that consistent adherence and persistence among patients with chronic conditions drop dramatically after the first 6 months of therapy [5, 24]. This may be particularly relevant in RA, where aggressive treatment in the early phases of the disease has been shown to prevent structural damage and to favor better outcomes, including a higher remission rate [25–28]. In such a clinical context, it may be intuitive to assume that earlier non-persistence will affect favorable outcomes, although non-persistence timing (early vs. late) did not appear to be a predictor. Interestingly, persistence duration was highly and negatively correlated with non-persistence timing (Spearman’s ρ = −0.8, *p* ≤ 0.001), which may explain the relevance of the former aspect of persistence over non-persistence timing. Finally, the cutoff at least 87.5 % of persistence duration to predict SR in the subpopulation of women highlights the patient’s need to adhere closely to prescriptions if major outcome benefits are desired. This cutoff is higher than the arbitrary categories of good and poor compliance, often set at 80 % [29], although that figure is based on a major and sustained outcome.

In addition to persistence duration, baseline DAS28, age and sex impacted the outcomes of interest.

Lower disease activity at RA diagnosis was found to be a predictor of subsequent disease activity, of SR and of function. Combe et al. [30] also found that patients with RA for whom therapy with one, two or three or more DMARDs had failed and who had higher disease activity at baseline were less likely to achieve remission after 6 months of golimumab. ten Kloster et al. [31] identified baseline predictors of achieving satisfactory improvement in pain in 209 ERAPs after 6 months of T2T therapy; among the predictors was 12 or fewer tender joints at baseline. Two additional studies performed in early RA confirmed a lower initial number of tender joints (which may be considered a surrogate of disease activity) as a predictor of remission [32, 33].

Age was found to be a predictor (in addition to disease activity) of SF. Data from two large inception cohorts identified that older age (in addition to other factors) was associated with increased likelihood of membership in subgroups with worse HAQ progression [34].

Young et al. [35] found that among 732 ERAPs, 9.4 % had marked functional loss at 5 years of follow-up, and this adverse functional outcome was more likely to occur in patients older than 60 years of age. Finally, the relationship between disability and demographic and clinical variables was analyzed in 684 patients with inflammatory polyarthritis referred to the Norfolk Arthritis Register. Older age at symptom onset (≥64 years) was one of the factors associated with disability as defined as HAQ of 1 or above [36].

Finally, female sex was an additional predictor of unfavorable outcome, as previously reported in other populations [37, 38]. Interestingly, women were highly represented in our cohort, as a female/male ratio of 7–8:1 has been described in Latin American surveys, which is well above the nearly 3:1 ratio reported in the United States and Europe [39].

The present study has some limitations. The first is that it is a single-center study, which potentially could decrease the generalizability of the results. Second, persistence was defined according to physician-reported and patient-reported discontinuation of treatment, and the threshold used to define non-persistence was arbitrarily chosen. Third, we analyzed the impact of a persistence construct on outcomes, but we did not examine the potential impact of a different construct such as adherence (i.e., compliance with) to medication. Fourth, we defined SF according as HAQ score of 20 or less. A different cutoff (0) on the HAQ-DI has been recommended on the basis that it may be (more) intuitive and sensitive and has face validity [40]. We repeated our analyses according to such suggestions and obtained similar results. Fifth, the study had a relatively short observation period of 5 years. Sixth, we did not include local controls in whom to assess the progression of functional disability largely explained by the ageing process [41]. Seventh, we did not include all the potential variables linked to worse disability, such as body mass index [42]. Finally, medication-taking behavior is a complex process in which frequency, characteristics and predictors may vary over the course of follow-up. Favorable and unfavorable disease courses have both been associated with adherence to therapy [12, 43], the latter reflecting, perhaps, the fact that active patients regard complex therapy as useless. Additional predictors are related to the disease itself, the population enrolled and the indicated therapy. In such a dynamic environment, the hypothetical causal chain of outcomes–adherence–outcomes is debatable and limits comprehensiveness of the topic.

## Conclusions

In the present study, we analyzed the impact of two particular aspects of the persistence construct—timing and duration—on RA outcomes. Persistence duration during the first 4 years of follow-up was a predictor of disease activity and disability in the following year, whereas the timing of non-persistence (earlier vs. late) was irrelevant. We also confirmed in our population additional predictors of favorable outcomes, such as younger age, male sex and lower disease activity at diagnosis.

## Abbreviations

ACCP: anti-cyclic citrullinated peptide antibodies
CI: confidence interval
CQ: Concordance Questionnaire
DAS28: Disease Activity Score in 28 joints
DMARD: disease-modifying anti-rheumatic drug
ERAP: early rheumatoid arthritis patient
HAQ: Health Assessment Questionnaire
HAQ-DI: Health Assessment Questionnaire disability index
OR: odds ratio
RA: rheumatoid arthritis
RF: rheumatoid factor
SD: standard deviation
SF: sustained function
SR: sustained remission
T2T: treat to target
